# New results on formaldehyde: the 2nd International Formaldehyde Science Conference (Madrid, 19–20 April 2012)

**DOI:** 10.1007/s00204-012-0966-4

**Published:** 2012-11-09

**Authors:** Hermann M. Bolt, Peter Morfeld

**Affiliations:** 1 Leibniz Research Centre on Working Environment and Human Factors (IfADo), TU Dortmund, Ardeystr. 67, 44139 Dortmund, Germany; 2 Institute and Policlinic for Occupational Medicine, Social Medicine and Social Hygiene, University of Cologne, Cologne, Germany; 3 Institute for Occupational Epidemiology and Risk Assessment of Evonik Industries, Essen, Germany

**Keywords:** Formaldehyde, Toxicity, Genotoxicity, Carcinogenicity, Epidemiology, Regulation

## Abstract

The toxicology and epidemiology of formaldehyde were discussed on the 2nd International Formaldehyde Science Conference in Madrid, 19–20 April 2012. It was noted that a substantial amount of new scientific data has appeared within the last years since the 1st conference in 2007. Progress has been made in characterisation of genotoxicity, toxicokinetics, formation of exogenous and endogenous DNA adducts, controlled human studies and epidemiology. Thus, new research results are now at hand to be incorporated into existing evaluations on formaldehyde by official bodies.

## Introduction

Triggered by evaluations of IARC (2006, 2012), the toxicity and carcinogenicity of formaldehyde are matters of current concern in the scientific and regulatory community. This issue has also been addressed in a recent Editorial of this journal (Bolt et al. 2010).

**Fig. 1:**
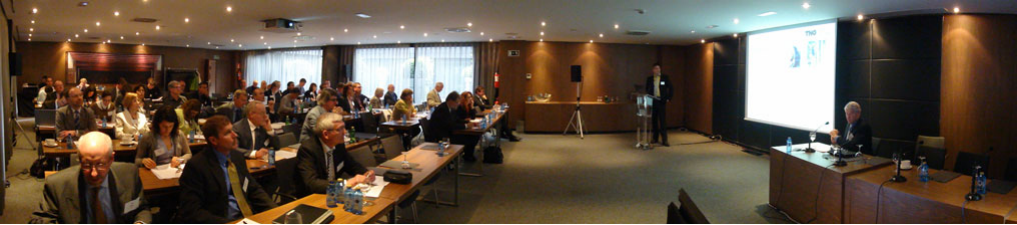
Audience of the 2nd International Formaldehyde Science Conference, Madrid, 19–20 April 2012. Copyright granted by CEFIC, Brussels

Five years after the 1st International Formaldehyde Science Conference (held in Barcelona 2007), academics and scientists met in Madrid on 19–20 April 2012, to debate the most recent scientific research data (Fig. 1). Thereby, the conference provided a forum for in-depth discussions of the current state on local and systemic effects of formaldehyde, as well as on associated regulatory aspects.

## Local and systemic effects of formaldehyde

The conference started with a state-of-the-art review by *Günter Speit* (Ulm) on genotoxicity of formaldehyde. In order to focus discussions at the conference, he addressed the following key theses: (1) formaldehyde is genotoxic in vitro, (2) formaldehyde mainly induces clastogenic effects in vitro, (3) formaldehyde induces genotoxic effects (DNA–protein cross-links) at the site of first contact with the organism, but is not passed to neighbour cells, (4) micronuclei at the site of first contact were not identified in exposed experimental animals, and findings in humans were considered as being inconclusive, (5) formaldehyde does not induce systemic genotoxic/mutagenic effects in animal experiments, (6) systemic and cytogenetic effects in human biomonitoring studies are likely not to be related to formaldehyde exposure.

*James A. Swenberg* (Chapel Hill, NC) presented new data of his group on exogenous and endogenous DNA adducts of inhaled formaldehyde and on epigenetic alterations of microRNAs. Exogenous formaldehyde leads to formation of DNA adducts identical to endogenous DNA adducts. Quantitative data are now available on endogenous adducts, and relation between endogenous and exogenous adducts. This includes quantitative data on endogenous and exogenous DNA adducts from the same samples (Swenberg et al. 2011). The use of stable isotopes (^13^C and ^2^H) allows differentiation of adducts formed from endogenous versus exogenous formaldehyde (Lu et al. 2012). It could be demonstrated that following exposure to formaldehyde exposure-induced DNA mono-adducts and cross-links only occur in nasal epithelial DNA of rats and primates. Only dG mono-adducts and cross-links are formed following inhalation to formaldehyde, whereas dA mono-adducts may arise from intracellular formation of formaldehyde secondary to intracellular metabolism or DNA–protein cross-links. Whereas endogenous DNA mono-adducts (dG and dA) are present in all cells and tissues, there is no exogenous formaldehyde adduct formation in bone marrow and other distant sites. It was concluded that this does not support a biological plausibility of formaldehyde-induced leukaemia.

## Biological monitoring

*Siegfried Knasmüller* (Vienna) reviewed biomonitoring studies using nasal and buccal cells for the detection of cytotoxic and DNA-damaging chemicals, including formaldehyde. About 300 micronuclei studies with exfoliated buccal cells and 19 studies with nasal cells were published so far, addressing lifestyle factors, health status, dietary factors and occupational exposures. Regarding formaldehyde, four further studies were published since the comprehensive review of Speit and Schmid (2006). An own evaluation revealed that two studies used Wright’s stain (a modified Giemsa staining procedure), considered to be non-specific. In one study, no micronuclei were found in controls, which is out of the normal range. Results of the remaining studies were controversial, and none of these fulfilled criteria for optimal investigations. Ladeira et al. (2011) and Viegas et al. (2010) conducted relatively large studies; they evaluated 2,000 cells but used Feulgen staining without counterstain. All other studies were smaller as suggested, and in some of them the number of evaluated cells was below 2,000. It was summarised that results obtained so far with formaldehyde-exposed individuals yielded controversial results; three studies in which positive results were recorded were considered inadequate due to methodological shortcomings.

*Carina Ladeira* (Lisbon) presented details of her own studies (Viegas et al. 2010; Ladeira et al. 2011), claiming a moderately positive correlation between micronucleus frequency in peripheral blood lymphocytes and the duration of formaldehyde exposure. In the following discussion, it was mentioned that a re-evaluation showed that repeated measurements of the same slide were highly variable not only between two scorers, but also when slides were evaluated by the same scorer (Speit et al. 2012).

*Roberto Bono* (Torino) investigated relationships between formaldehyde exposure of technicians of pathology wards, workers of plastic laminates industry and controls, with the alkylation of haemoglobin to form terminal *N*-methylenevaline residues. Special emphasis was laid on examination of smoking habits. A positive correlation was demonstrated between exposure to formaldehyde in the occupationally exposed groups and haemoglobin alkylation to form *N*-methylenevaline. Tobacco smoking had an only minor impact on the formation of this adduct (Santovito et al. 2011; Bono et al. 2012).

*Anne Kleinnijenhuis* (Zeist) elaborated a new HPLC/MS-based method for analysis of formaldehyde in blood, also employing a differentiation between endogenous and exogenous formaldehyde by use of ^13^C-labelling. This was applied to an experimental study in rats, with samplings during and after inhalation exposure to formaldehyde (10 ppm for 6 h). There was no increase in blood formaldehyde during this exposure. It was, therefore, concluded to be unlikely that inhaled formaldehyde at this dose level entered the blood to cause leukaemia.

## Systemic effects of formaldehyde in humans?

*Simone Neuss* (Ludwigshafen) reported on formaldehyde-induced gene expression changes in humans and in vitro. As part of a larger experimental human study (Mueller et al. 2012), in which volunteers (male non-smokers) were exposed to formaldehyde for 4 h/day over a period of 5 days between 0 and 0.7 ppm, blood samples, exfoliated nasal mucosa cells and nasal biopsies were taken. The mRNA expression of formaldehyde dehydrogenase (FDH, alcohol dehydrogenase 5, EC 1.2.1.46) was measured in blood samples by quantitative real-time reverse transcription polymerase chain reaction with TaqMan probes. DNA microarray analyses using a full-genome human microarray were performed on blood samples and nasal biopsies of selected subgroups with the highest formaldehyde exposure at different days. Inhalation of formaldehyde did also not cause alterations in the expression of genes in a microarray analysis with nasal biopsies and peripheral blood cells.

*Luoping Zhang* (Berkeley, CA) discussed potential systemic effects of formaldehyde and underlying possible mechanisms. Arguments of this kind had been relevant for the IARC (2012) evaluation of sufficient evidence that formaldehyde causes leukaemia, particularly of the myeloid type. A meta-analysis (Zhang et al. 2009) investigated the coherence of formaldehyde exposure and blood neoplasia and pointed to an increased incidence of myeloid leukaemia in occupationally exposed persons. As mechanistic explanation, alternative models were presented: interaction of formaldehyde with human haematopoietic stem cells circulating in the blood and/or interaction of formaldehyde with primitive pluripotent stem cells in nasal/oral passages. A third possibility could be that formaldehyde could get to the bone marrow via methanediol (the hydrated form of formaldehyde). Although it was considered that the mode of action in humans is largely unknown, it was claimed that (1) haematotoxicity (reduced blood cell counts) was induced in formaldehyde-exposed workers, pointing to bone marrow toxicity, and formaldehyde-inhibited myeloid progenitor (CFU-GM) colony formation, (2) myeloid leukaemia-related chromosomal aneuploidy (−7, +8) was detected in haematopoietic stem/progenitor cells circulating in the blood of formaldehyde-exposed humans, (3) potential modes of action included DNA/chromosomal damage, DNA repair failure and growth advantage of formaldehyde-damaged human stem cells, (4) formaldehyde was reviewed to induce reproductive and developmental toxicity as systemic effects (updated meta-analysis, Dong et al. 2011). It was postulated that more and larger human studies should be performed to confirm the current results and their understanding.

*Stephanie Kuehner* (Ulm) reported on a study that was performed in response to the suspicion of induction of leukaemia by formaldehyde. Chromosome preparations from cultured myeloid progenitor cells were obtained from blood samples of healthy subjects. These were analysed by fluorescence in situ hybridization (FISH) for spontaneously occurring numerical aberrations after cultivation for 9 days. FISH analysis with probes for chromosomes 6, 7 and 8 revealed that the baseline frequency of aneuploid metaphases was similar and low for the three chromosomes tested. Also, myeloid progenitor cells were exposed during the cultivation period to formaldehyde, and the frequency of aneuploidies after 9 days of cultivation was determined. The results indicated that formaldehyde did not induce aneuploidy under these conditions. It was seen that myeloid progenitor cells from healthy subjects were not particularly sensitive towards the cytotoxic action of formaldehyde. Also with other cell lines a significant aneugenic action could not be established by the cytokinesis block micronucleus test, which was further supported by gene expression studies (signatures of clastogens/mutagens). The results were interpreted not to support the assumption of a specific effect of formaldehyde on myeloid progenitor cells (Kuehner et al. 2012).

*Frieke Kuper* (Zeist) presented a retrospective examination of archived nasal epithelial lymphoid tissues (NALT) of rats and mice from earlier chronic formaldehyde studies at Battelle, Columbus, OH (exposures between 2 and 15 ppm; Kerns et al. 1983). She outlined that evaluation of such tissues is difficult, and that NALT is rarely examined in inhalation toxicity studies. Enhanced histopathology was, therefore, performed on the archived material. Application of the proliferation marker bromo-deoxyuridine (28 days inhalation study in rats and mice) showed epithelial hyperplasia of the lympho-epithelium of NALT in rats repeatedly exposed to the tumour-inducing concentration of 15 ppm formaldehyde. No effect was seen on local lymphoid tissue of mice.

## Formaldehyde epidemiology and cancer risk

*Craig Steinmaus* (Berkeley, CA) discussed a previously published meta-analysis focusing on high-exposure groups and myeloid leukaemia. The analysis included two large studies in particular: one involving >25,000 workers in US formaldehyde industries and the other involving a cohort of >13,000 funeral directors and embalmers. Formaldehyde was found associated with increased risks of leukaemia (relative risk = 1.53; 95 % confidence interval = 1.11–2.21; *p* = 0.005; 14 studies), specifically myeloid leukaemia (relative risk = 2.47; 95 % confidence interval = 1.42–4.27; *p* = 0.001; 4 studies). This analysis was seen to provide evidence of an increased myeloid leukaemia risk with high exposures to formaldehyde (Schwilk et al. 2010).

*Peter Morfeld* (Cologne) critically reviewed and evaluated the epidemiology on formaldehyde exposure and leukaemia risk. Modelling problems and missing data complicated interpretations of the recent study on embalmers by Hauptmann et al. (2009). In contrast to the meta-analysis by Bachand et al. (2010) who reported an unexceptionable relative risk, Schwilk et al. (2010) identified statistically significant elevations. The difference between both summaries was not caused by study selection but by the specific focus on high-exposure subgroups in Schwilk et al. (2010). Morfeld noted that the latter approach, although increasing the power to detect an excess risk, is rather unusual and suffers from methodological shortcomings: The analysis did not make use of all information available; highest exposure cut points and exposure metrics varied across studies (e.g. duration, peak exposure, cumulative exposure). This may have caused a relevant heterogeneity between the combined study results. In such a situation, predictive intervals are recommended instead of calculating the usual confidence intervals (Riley et al. 2011; Graham and Moran 2012). The finding of elevated leukaemia risks was no longer significant when using these statistical techniques and the apparent conflict to Bachand et al. (2010) could be resolved.

*Gary Marsh* (Pittsburgh, PA) reported on the formaldehyde epidemiology of nasopharyngeal cancers (NPC). The most relevant epidemiological investigation is an industrial cohort study performed by the National Cancer Institute (NCI) indicating an NPC excess risk after formaldehyde exposure (Hauptmann et al. 2004). However, due to the low number of cases and the clustering of theses cases in just one out of ten plants, no consistent evidence could be obtained (Marsh and Youk 2005; Marsh et al. 2007b). Additional analyses of the critical plant subcohort indicated a possible association of NPC risk with prior work in the metal industry (Marsh et al. 2007a). Other cohort studies did not support the association (Coggon et al. 2003; Pinkerton et al. 2004). Marsh reported on an incomplete mortality follow-up of the NCI cohort study that resulted into biased estimates overstating the true risks (Beane Freeman et al. 2009). Unfortunately, no updated and corrected estimates of the NPC risks are available yet (Marsh et al. 2010).

## Exposure limits and susceptible populations

On the basis of a comprehensive compilation of formaldehyde in the indoor environment (Salthammer et al. 2011), *Tunga Salthammer* (Hannover) presented an overview on the technical indoor sources of formaldehyde, available emission data and the regulation of formaldehyde indoor levels, internationally. It was outlined that in the indoor environment, formaldehyde concentrations have been continuously decreasing, down to current levels of 20–40 ppb. This may contrast to trends of increasing outdoor concentrations. He expressed doubt whether indoor guidelines in the range of 10 ppb were needed and sustainable. From a technical standpoint, a justifiable balance between costs and benefit should be observed, and support was expressed to the current WHO indoor guidance value (see below).

*Gunnar Nielsen* (Copenhagen) pointed out that the exposure–response relationship for nasal cancer in rats is highly nonlinear, supporting a no-observed-adverse-effect level (NOAEL) that allows deriving a health-based guideline value. Departing from the classical rat studies, an indoor guideline value of 0.08 ppm (0.1 mg/m^3^) formaldehyde was considered by WHO (2000) as being preventive of carcinogenic effects, also in compliance with epidemiological findings. The main reasoning was as follows (Nielsen and Wolkoff 2010; Wolkoff and Nielsen 2010): (1) Epidemiological studies reported no increased incidence of nasopharyngeal cancer in humans below a mean exposure level of 1 ppm and peak levels below 4 ppm, consistent with results from rat studies. (2) Rat studies indicated that cytotoxicity-induced cell proliferation (NOAEL at 1 ppm) is a key mechanism in the development of nasal cancer. (3) Lympho-haematopoietic malignancies are not consistently observed in animal studies and, if caused by formaldehyde in humans, are considered to be high-dose phenomena with nonlinear exposure–response relationships. Such diseases were not reported in epidemiological studies at peak exposures below 2 ppm and average exposures below 0.5 ppm. In rodents, the nasal cancer effect of formaldehyde is much more prominent than lympho-haematopoietic malignancies. Thus, exposure limits preventing nasal cancer were also considered to prevent lympho-haematopoietic malignancies.

*Hermann M. Bolt* (Dortmund) explained the strategy of the Scientific Committee on Occupational Exposure Limits (SCOEL) of the EU with respect to carcinogens in general (Bolt and Huici-Montagud 2008) and to formaldehyde in particular (SCOEL 2008). SCOEL has recommended an Occupational Exposure Limit for formaldehyde of 0.2 ppm, based on a carcinogenic threshold mechanism and the avoidance of any sensory irritation in exposed humans. The overall argumentation was similar to that of WHO (2000) for indoor limits and was based on the studies available in 2008.

*Gerhard Triebig* (Heidelberg) presented a new exposure study in volunteers to examine chemosensory effects of formaldehyde in hyposensitive and hypersensitive persons (Mueller et al. 2012). Forty-one male volunteers were exposed for 5 days (4 h per day) in a randomised schedule to the control condition (0 ppm) and to formaldehyde concentrations of 0.5 and 0.7 ppm and to 0.3 ppm with peak exposures of 0.6 ppm, and to 0.4 ppm with peak exposures of 0.8 ppm, respectively. Peak exposures were carried out four times a day over a 15-min period of time. Subjective pain perception induced by nasal application of carbon dioxide served as indicator for sensitivity to sensory nasal irritation. The following parameters were examined: subjective rating of symptoms and complaints (Swedish Performance Evaluation System), conjunctiva redness, eye-blinking frequency, self-reported tear film break-up time and nasal flow rates. The influence of personality factors on the volunteer’s subjective scoring was examined (positive and negative affect schedule). Formaldehyde exposures to 0.7 ppm for 4 h and to 0.4 ppm for 4 h with peaks of 0.8 ppm for 15 min caused no significant sensory irritation of the measured conjunctiva and nasal parameters. No differences between hypo- and hypersensitive subjects were seen. Based on this study, it was concluded that formaldehyde concentrations of 0.7 ppm for 4 h and of 0.4 ppm for 4 h with peaks of 0.8 ppm for 15 min did not cause adverse effects related to irritation, and that no differences between hypo- and hypersensitive subjects were observed.

The study was accompanied by satellite investigations (Zeller et al. 2011a, b, 2012). *Günter Speit* (Ulm) reported that the 41 volunteers were also tested for susceptibility towards unspecific nasal irritation (sensitivity towards CO_2_), in order to define subgroups of “hypersensitive” and “hyposensitive” subjects. The results indicated that despite large differences in CO_2_ sensitivity, the susceptibility towards nasal irritation was not related to the induction of genotoxic effects (DPX, SCE) in peripheral blood or to the protection of blood cells against formaldehyde-induced effects (expression of FDH, repair capacity for FA-induced DPX). There was no correlation between CO_2_ sensitivity and the expression of FDH. There was also no close correlation between the various indicators of cellular sensitivity towards FA-induced genotoxic effects, and no subgroups were identified with particular mutagen sensitivity towards formaldehyde (Zeller et al. 2011a). Moreover, investigations of potential individual susceptibility of human blood cells towards formaldehyde-induced genotoxicity indicated no biologically relevant differences with regard to various indicators of cellular sensitivity to genotoxic effects along with the expression of formaldehyde dehydrogenase and genetic polymorphisms of the glutathione S-transferases *GSTT1* and *GSTM1*. None of the different study groups showed particular mutagenic sensitivity to formaldehyde (Zeller et al. 2012). It was concluded that a low scaling factor to address possible human inter-individual differences in formaldehyde-induced genotoxicity could be reasonable.

## Round table discussion

The conference ended with a round table discussion on implications of the new findings for the scientific derivation of exposure limits. It was concluded that a substantial body of new data has been published on formaldehyde toxicology and epidemiology, particularly in the years 2010–2012, as evidenced by the references list of this meeting report. Such new data should now be incorporated into existing evaluations.

In summary, the conference successfully updated the scientific discussions on the health effects after formaldehyde exposure. Such substance-specific conferences are highly welcome, because these facilitate an exchange between scientists internationally to discuss most recent developments in toxicology and epidemiology, concentrating on one substance only. Larger scientific conferences are usually unable to provide enough time on in-depth discussions and controversies related compound-specific questions. This conference reserved enough time for both presentation and debate.

The presentation slides are available online via http://www.formacare.org/about-formaldehyde/science/formaldehyde-science-conference
